# An AAV-CRISPR/Cas9 strategy for gene editing across divergent rodent species: Targeting neural oxytocin receptors as a proof of concept

**DOI:** 10.1126/sciadv.adf4950

**Published:** 2023-05-31

**Authors:** Arjen J. Boender, Marina Boon, H. Elliott Albers, Samantha R. Eck, Brandon A. Fricker, Aubrey M. Kelly, Joseph E. LeDoux, Simone C. Motta, Prerana Shrestha, Jack H. Taylor, Brian C. Trainor, Rodrigo Triana-Del Rio, Larry J. Young

**Affiliations:** ^1^ Center for Translational Social Neuroscience, Silvio O. Conte Center for Oxytocin and Social Cognition, Emory National Primate Research Center, Emory University, Atlanta, GA, USA.; ^2^ Department of Human Genetics, Donders Institute for Brain, Cognition and Behavior, Radboud University Medical Center, Nijmegen, Netherlands.; ^3^ Neuroscience Institute, Georgia State University, Atlanta, GA, USA.; ^4^ Center for Behavioral Neuroscience, Georgia State University, Atlanta, GA, USA.; ^5^ Department of Psychology, University of California, Davis, Davis, CA, USA.; ^6^ Department of Psychology, Emory University, Atlanta, GA, USA.; ^7^ Center for Neural Science, New York University, New York, NY, USA.; ^8^ Department of Psychiatry and Department of Child and Adolescent Psychiatry, New York University Langone Medical School, New York, NY, USA.; ^9^ Institute of Biomedical Sciences, Department of Anatomy, University of São Paulo, São Paulo, SP, Brazil.; ^10^ Department of Neurobiology and Behavior, Renaissance School of Medicine, Stony Brook University, Stony Brook, NY, USA.; ^11^ Department of Psychiatry and Behavioral Sciences, Emory University School of Medicine, Atlanta, GA, USA.

## Abstract

A major issue in neuroscience is the poor translatability of research results from preclinical studies in animals to clinical outcomes. Comparative neuroscience can overcome this barrier by studying multiple species to differentiate between species-specific and general mechanisms of neural circuit functioning. Targeted manipulation of neural circuits often depends on genetic dissection, and use of this technique has been restricted to only a few model species, limiting its application in comparative research. However, ongoing advances in genomics make genetic dissection attainable in a growing number of species. To demonstrate the potential of comparative gene editing approaches, we developed a viral-mediated CRISPR/Cas9 strategy that is predicted to target the oxytocin receptor (*Oxtr*) gene in >80 rodent species. This strategy specifically reduced OXTR levels in all evaluated species (*n* = 6) without causing gross neuronal toxicity. Thus, we show that CRISPR/Cas9-based tools can function in multiple species simultaneously. Thereby, we hope to encourage comparative gene editing and improve the translatability of neuroscientific research.

## Teaser

The development of comparative gene editing strategies improves the translatability of animal research.

## INTRODUCTION

Nature provides an abundant variety of organisms using neural systems to interact with their environment to ensure survival and maximize fitness in diverse ways (*1*). Comparative neuroscience takes advantage of this diversity among species to find general principles of neural circuit functioning as well as mechanisms giving rise to variation in neural function and behavior (*2*). One efficient method to investigate the functional role of neural circuits is genetic dissection, a family of molecular approaches that allows for the manipulation of targeted genes (e.g., gene knockdown or exogenous expression) in specific cell populations (*3*–*5*). Traditionally, genetic dissection has been tractable in only a handful of model species, most commonly in laboratory mice, and this has limited our ability to leverage this powerful comparative approach to help improve treatments for neurobehavioral psychiatric disorders (*6*). However, ongoing efforts to construct whole-genome assemblies of nontraditional model species (*7*), along with the recent development of efficient genome editing tools (*8*, *9*), which can be used in conjunction with versatile viral vectors (*10*), have made it increasingly feasible to use genetic dissection strategies in a wide range of species (*11*–*15*). We believe that the application of genetic dissection approaches across a range of species has great fundamental and translational value, as it will greatly enhance our ability to take advantage of nature’s remarkable diversity to bolster our understanding of species-specific and general mechanisms of complex brain-behavior relationships. Here, we demonstrate the feasibility of designing viral-mediated CRISPR/Cas9 tools for targeted gene editing across a wide range of model organisms. While we focus on neural oxytocin receptors (OXTRs) in rodents, this strategy should be effective for targeting any gene in any tissue across model organisms.

As a proof of concept, we developed a strategy for disrupting OXTR signaling in a wide range of rodents, as diverse members of this order are used in OXT research, particularly in relation to social behavior (*16*–*22*). OXT is a highly conserved peptide that has been studied in many species (*14*, *23*–*27*), and this research has highlighted its role in the regulation of numerous social behaviors, including parental care, social bonding, mate preference, social recognition, and social vigilance (*28*, *29*). The OXT system also has translational potential as a target for psychiatric disorders with disruptions in the social domain (*30*–*32*). A remarkable feature of the oxytocinergic system is that *Oxt* expression patterns are highly conserved across species, with *Oxt* being expressed primarily in paraventricular and supraoptic hypothalamic nuclei across vertebrates (*33*). This is in sharp contrast to its receptor (*Oxtr*), which shows strong intra- and interspecific variation even among closely related species (*28*, *29*, *34*, *35*). Variation in OXTR density in various brain regions affects the processing of social information, which is thought to contribute to diversity in social behaviors (*20*, *28*, *36*). To be able to explore OXTR function from a comparative perspective, tools are needed to enable the genetic dissection of OXTR signaling in multiple species. While systemic *Oxtr* knockout lines exist for a few species (*13*, *14*, *23*, *37*), a single, cross-species tool to robustly disrupt functional OXTR signaling using targeted gene disruption in a spatially and temporally controlled manner in multiple rodent species has not been available. The development of such a tool is valuable to the comparative neuroscience field as it negates the need for extensive validation in each individual species. It will facilitate the applicability of gene editing in species that are studied for their interesting behavior, but for which genetic tools are not readily available, and thereby diversify the investigation of behaviors that are known to be modulated by OXT, but are not easily studied in mice (e.g., biparental care, pair bonding, partner loss, and female aggression) (*15*, *20*, *35*, *38*).

Viral-mediated CRISPR/Cas9 genome editing has emerged as an efficient and versatile strategy to induce indels (insertions/deletions) in protein-coding sequences and perturb associated functional protein levels (*11*). We adopted an adeno-associated virus (AAV)–based strategy to deliver the CRISPR/Cas9 components to adult neural tissue (*10*) and reduce OXTR protein levels in vivo. The system consists of a Cas9 endonuclease from *Streptococcus pyogenes* (spCas9) that induces double-strand breaks in the DNA, and a guide RNA (gRNA), whose specificity determines the genomic location of Cas9-mediated mutagenesis. By targeting regions in the *Oxtr* coding sequence that are conserved across rodent species, we demonstrated the efficacy of this AAV-CRISPR/Cas9 vector at reducing OXTR density in six rodent species used in behavioral neuroendocrine research, and we predict that this approach will be effective in more than 80 rodent species. Furthermore, we demonstrated selectivity of the AAV-CRISPR/Cas9 approach by assessing its effect on the expression of a close match in the rodent genome, the arginine vasopressin receptor 1A (*Avpr1a*). Thereby, this tool will facilitate the comparative study of OXT signaling and advance our understanding of general principles of this ancient neuropeptide’s function as well its role in modulating a diverse range of social behaviors in a species-specific manner. These results also provide a proof of principle for a strategy that can be widely applied to other genes in comparative neuroscience research.

## RESULTS

### Design of viral strategy to target *Oxtr* in multiple rodents

**Fig. 1.**
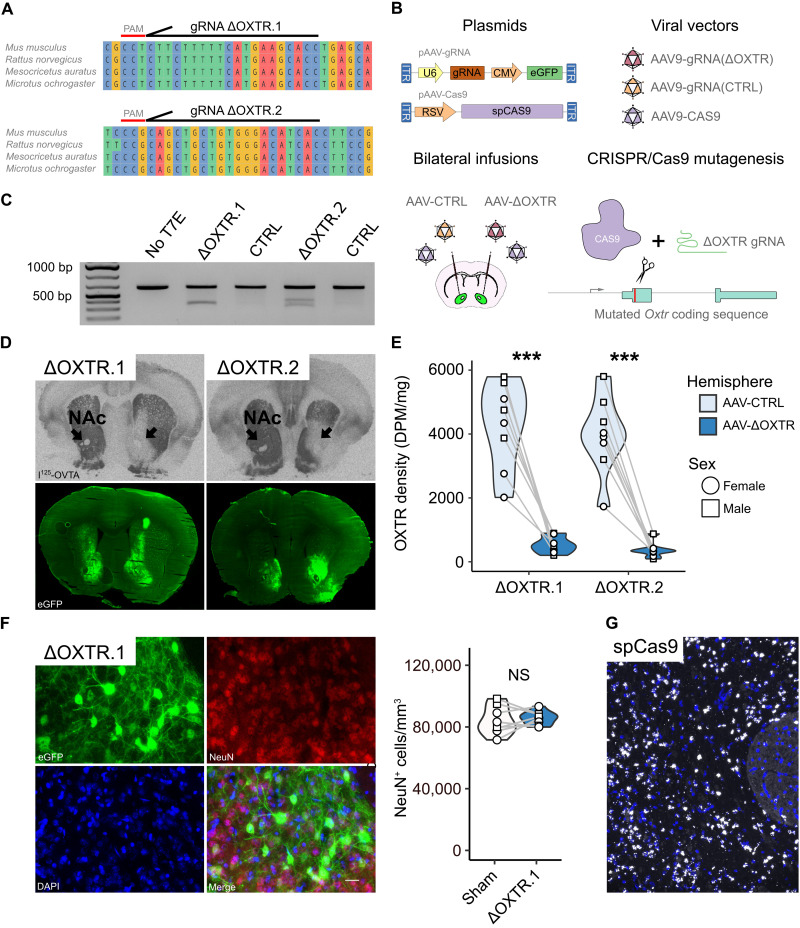
Design of AAV-CRISPR/Cas9 strategy to functionally perturb OXTR signaling in multiple species and its validation in the prairie vole. (**A**) Schematic depicting the two conserved regions in the *Oxtr* coding sequence in four rodent species that are amenable to CRISPR/Cas9–mediated mutagenesis. (**B**) Schematics illustrating the major elements within the AAV plasmids, the AAV particles and how they are infused in the brain, as well as how the CRISPR/Cas9 technique functions. (**C**) Image of agarose gel evidencing gene editing after infusion of AAV-ΔOXTR, but not AAV-CTRL. (**D**) Top images are representative I^125^-OVTA autoradiograms of brain sections of AAV-CRISPR/Cas9–injected prairie voles, and bottom images are adjacent brain sections that show viral-induced eGFP fluorescence. Black arrows indicate the target area. (**E**) Quantification of OXTR levels in AAV-ΔOXTR–injected hemispheres and AAV-CTRL–injected hemispheres. Paired *t* tests, ΔOXTR.1: *N* = 8, ***P = 0.0001; ΔOXTR.2: *N* = 8, ***P = 4.36 × 10^−5^. (**F**) Images of native eGFP fluorescence, immunofluorescence of NeuN protein, and 4′,6-diamidino-2-phenylindole (DAPI) staining in AAV-ΔOXTR–infected tissue on the left and quantification of Neun^+^ cells in AAV- and sham-injected tissue. Paired *t* test: *N* = 8, *P* = 0.6. Scale bar, 20 μm. NS, nonsignificance. (**G**) Representative image of *spCas9* mRNA expression in the prairie vole. Scale bar, 100 μm.

Our goal was to develop a viral vector–mediated tool to reduce OXTR density in brains across a wide range of rodent model species. We aimed to validate the efficacy of our tool using six model species that are used in OXT research: *Acomys cahirinus* (spiny mouse), *Mesocricetus auratus* (golden hamster), *Microtus ochrogaster* (prairie vole), *Mus musculus* (house mouse), *Peromyscus californicus* (California deer mouse), and *Rattus norvegicus* (Norway rat). We previously developed an AAV-CRISPR/Cas9 strategy using a gRNA targeting the prairie vole *Oxtr* to efficiently reduce functional OXTR in the brain (*11*, *39*); however, the gRNA target sequence was not conserved across rodent species. Here, we used a similar approach using gRNAs targeting *Oxtr* sequences that are conserved across rodent species. To identify conserved *Oxtr* coding sequences that were amenable for mutagenesis by AAV-CRISPR/Cas9, we used the ClustalW algorithm of the msa package in R/Bioconductor to align the *Oxtr* coding sequences of species with available RefSeq sequence data (golden hamster, house mouse, Norway rat, and prairie vole) (*40*). Two conserved regions were found to be of sufficient length for gRNA targeting (>19 nucleotides) and to contain a permissive protospacer adjacent motif (PAM) sequence (5′-NGG-3′) (Fig. 1A). The gRNA(ΔOXTR)s target sequences in or just before the second transmembrane domain.

### Validation of the viral strategy in the prairie vole

We produced AAV-gRNA(ΔOXTR) vectors to target these two regions and first assessed their effectiveness and specificity in the prairie vole. AAV-gRNA(ΔOXTR) vectors were combined with AAV-Cas9 vectors and injected unilaterally in the nucleus accumbens (NAc), while the contralateral side received an AAV-CTRL mix (Fig. 1B). The gRNA(CTRL) targets a sequence of the bacterial *LacZ* gene, which is not expressed in vertebrates. The NAc expresses high levels of *Oxtr* in prairie voles and is thought to mediate monogamous pair bonding behaviors (*41*). Two weeks after surgery, AAV-CRISPR/Cas9–mediated genomic editing was validated through a T7 endonuclease assay (Fig. 1C). DNA was isolated from enhanced green fluorescent protein (eGFP)–positive tissue (Fig. 1D), and the AAV-CRISPR/Cas9–targeted *Oxtr* coding regions were polymerase chain reaction (PCR)–amplified. T7 endonuclease restriction was observed in PCR amplicons, which were of the expected length from AAV-ΔOXTR–infected tissue, but not in AAV-CTRL–infected tissue, indicating that specific mutagenesis had occurred in the *Oxtr* coding sequence. The digested fragments were of the expected size based on the position of the gRNA target sequence. To test whether these mutations translated into reduced OXTR protein levels, we performed I^125^-OVTA autoradiography on brain tissue (Fig. 1, D and E). Both AAV-gRNA(ΔOXTR) vectors strongly reduced I^125^-OVTA binding in the NAc, indicating that the generated *Oxtr* indels disrupted functional OXTR production. Reduction in OXTR levels was not caused by neuronal cell death because AAV-ΔOXTR–injected and sham-injected hemispheres contained a similar number of cells that were positive for the neuronal marker NeuN. In addition, we performed RNAscope in situ hybridization and found faithful expression of *spCas9* in AAV-injected hemispheres (*N* = 2) (Fig. 1G).

### The viral strategy does not affect AVPR1A levels in the prairie vole

**Fig. 2.**
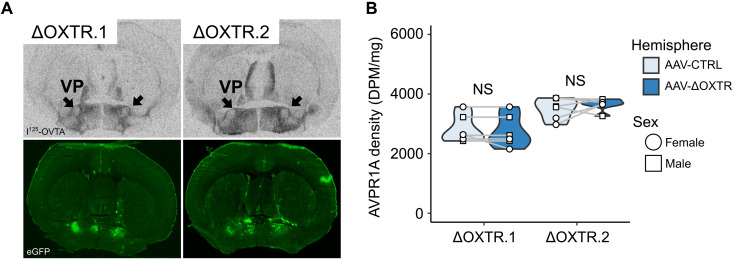
AAV-ΔOXTR does not affect AVPR1A levels in the prairie vole ventral pallidum. (**A**) Top images are representative I^125^-AVP autoradiograms of brain sections of AAV-CRISPR/Cas9–injected prairie voles, and bottom images are adjacent brain sections that show viral-induced eGFP fluorescence. Black arrows indicate the target area (VP, ventral pallidum). (**B**) Quantification of OXTR levels in AAV-ΔOXTR–injected hemispheres and AAV-CTRL–injected hemispheres. Paired *t* tests, ΔOXTR.1: *N* = 6, *P* = 0.37; ΔOXTR.2: *N* = 6, *P* = 0.38.

Then, we sought to assess potential off-target effects of the gRNA(ΔOXTR)s. The vasopressin receptor 1A (*Avpr1a*) and vasopressin receptor 1B (*Avpr1b*) genes are closely related to *Oxtr* and share high sequence homology (*42*). While both gRNA target sequences do not align to the prairie vole *Avpr1b* sequence [>10–base pair (bp) mismatches], the gRNA(ΔOXTR.2) target sequence has the highest sequence homology to *Avpr1a* (3-bp mismatches) of all genes, which makes it the most likely off-target gene in the prairie vole genome for this gRNA. The gRNA(ΔOXTR.1) target sequence differs from the prairie vole *Avpr1a* sequence by 8 bp. To functionally assess the specificity of the gRNAs, we used the injection strategy as described above and targeted a region in the prairie vole in which *Avpr1a* is expressed: the ventral pallidum. AVPR1A autoradiography revealed no disruption in AVPR1A levels in the targeted area, demonstrating the selectivity of our gRNA(ΔOXTR)s (Fig. 2).

### Efficacy of the viral strategy in five more rodent species

**Fig. 3.**
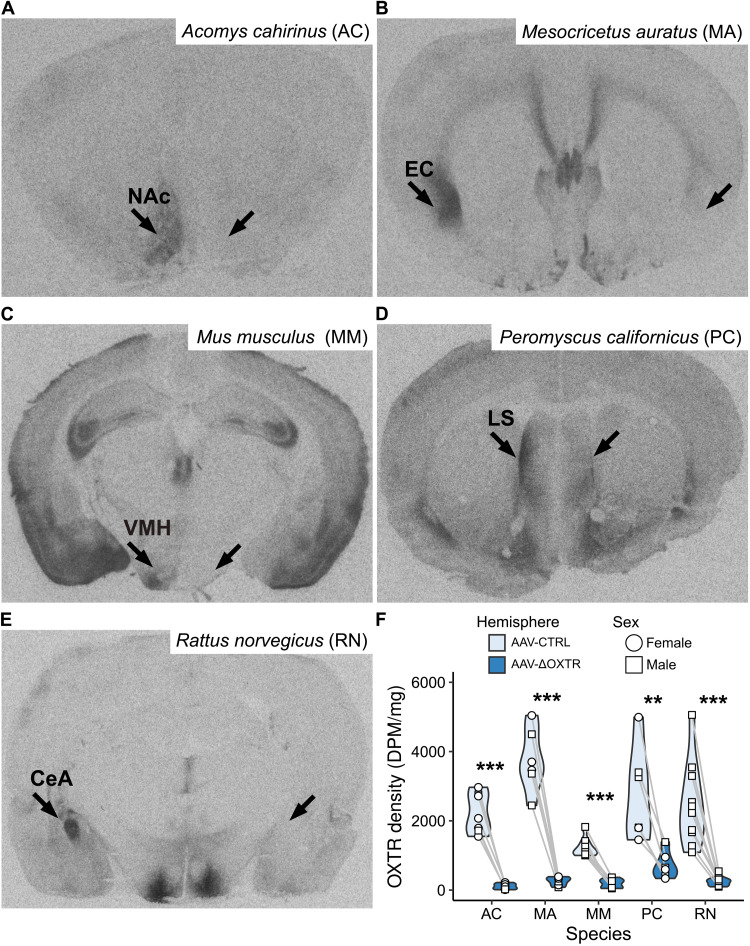
AAV-ΔOXTR reduces OXTR density in five more rodent species. Representative images of I^125^-OVTA autoradiograms of AAV-CRISPR/Cas9–injected (**A**) spiny mouse (AC), (**B**) golden hamster (MA), (**C**) house mouse (MM), (**D**) California deer mouse (PC), and (**E**) Norway rat (RN). (**F**) Quantification of OXTR levels in AAV-ΔOXTR–injected hemispheres and AAV-CTRL–injected hemispheres per species. Paired *t* tests: AC: *N* = 7, ***P = 4.57 × 10^−5^; MA: *N* = 6, ***P = 0.0001; MM: *N* = 7, ***P = 4.1 × 10^−5^; PC: *N* = 6, **P = 0.006; RN: *N* = 10, ***P = 0.0001. Black arrows depict the target areas (NAc, nucleus accumbens; EC, endopiriform cortex; VMH, ventromedial hypothalamus; LS, lateral septum; CeA, central amygdala).

Next, we tested the efficacy of AAV-gRNA(ΔOXTR.1) in five more rodent species (Fig. 3). As OXTR expression varies extensively among species, a range of brain areas was selected for injection based on the distribution of OXTR in each species (*43*–*45*). The NAc was targeted in spiny mice, the endopiriform cortex in golden hamsters, the ventromedial hypothalamus (VMH) in house mice, the lateral septum in California deer mice, and the central amygdala (CeA) in Norway rats. AAV-gRNA(ΔOXTR.1) significantly reduced OXTR levels across species and areas. In spiny mice, however, the efficacy was lower than in other species (fig. S1). During the preparation of this manuscript, the genome assembly of spiny mice became available, and we found no PAM sequence next to the target sequence of gRNA(ΔOXTR.1), which could explain the decreased effectiveness of gRNA(ΔOXTR.1) in spiny mice. However, the gRNA(ΔOXTR.2) target sequence is located next to a PAM site, so we injected a second batch of animals with this vector and observed strongly reduced OXTR levels. In sum, for all species, we achieved a significant reduction in OXTR levels.

### The viral strategy targets a wide range of rodent species

**Fig. 4.**
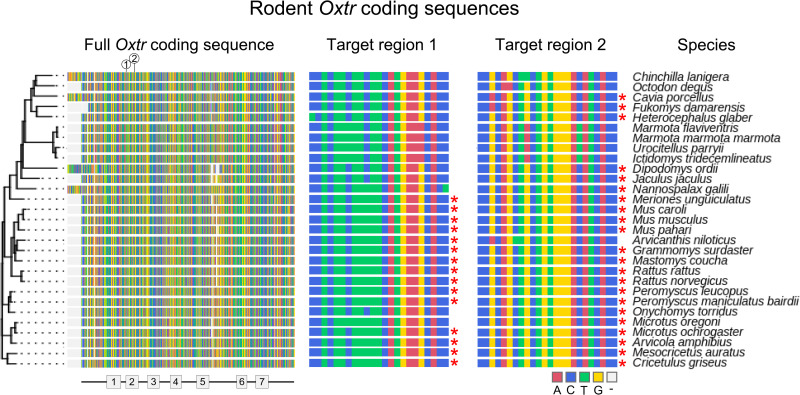
Alignment of all current rodent RefSeq *Oxtr* coding sequences and their match to the gRNA target sequences. Depicted on the left is a multiple sequence alignment of all available RefSeq rodent *Oxtr* coding sequences (*N* = 30). The labels on top indicate the position of the gRNA target sequences, and the labels on the bottom indicate the transmembrane portions of the protein. In the middle, the gRNA target regions are shown. A red asterisk next to the sequence indicates perfect alignment with the gRNA sequence. Scientific names are shown on the right.

We subsequently used the blastn algorithm from the National Center for Biotechnology Information (NCBI) BLAST+ software suite to search available whole-genome data and identify species in which either of the two gRNA(ΔOXTR) target sequences aligns perfectly and is thus predicted to work (*46*). We first aligned all available rodent RefSeq *Oxtr* sequences (*N* = 30) and found gRNA(ΔOXTR.1) to be functional in 15 species and gRNA(ΔOXTR.2) to be functional in 23 species (Fig. 4). Next, we searched for perfect alignment of the gRNA target sequences in all publicly available rodent genomes (*N* = 231). In this search, we found gRNA(ΔOXTR.1) to perfectly align in 43 species, and gRNA(ΔOXTR.2) in 80 species. Together, these viral vectors are likely to function in at least 81 rodent species (Table 1). Of note is that most of the rodent genomes are not annotated (e.g., spiny mouse and California deer mouse), so a perfect alignment does not necessarily indicate that the *Oxtr* coding sequence is targeted, and could indicate genes that closely resemble *Oxtr*, such as the vasopressin receptor genes. However, if interested in using this tool in a species for which no annotated genome is available, one could determine the sequence homology of the regions surrounding the gRNA target sequence with other *Oxtr* sequences to ensure specific targeting of the *Oxtr* gene.

**Table 1.** List of rodent species that contain gRNA(ΔOXTR) target sequences in their genome.

Species	gRNA(ΔOXTR.1)	gRNA(ΔOXTR.2)
*Acomys cahirinus*	No	Yes
*Acomys russatus*	No	Yes
*Apodemus speciosus*	No	Yes
*Apodemus sylvaticus*	No	Yes
*Arvicanthis niloticus*	Yes	No
*Arvicola amphibius*	Yes	Yes
*Capromys pilorides*	No	Yes
*Cavia aperea*	No	Yes
*Cavia porcellus*	No	Yes
*Cavia tschudii*	No	Yes
*Cricetomys gambianus*	No	Yes
*Cricetulus griseus*	Yes	Yes
*Dasyprocta punctata*	No	Yes
*Dinomys branickii*	No	Yes
*Dipodomys ordii*	No	Yes
*Dipodomys spectabilis*	No	Yes
*Dipodomys stephensi*	No	Yes
*Dolichotis patagonum*	No	Yes
*Ellobius lutescens*	Yes	Yes
*Ellobius talpinus*	Yes	Yes
*Erethizon dorsatum*	No	Yes
*Fukomys damarensis*	No	Yes
*Glis glis*	No	Yes
*Grammomys surdaster*	Yes	Yes
*Heterocephalus glaber*	No	Yes
*Hydrochoerus hydrochaeris*	No	Yes
*Hylomyscus alleni*	Yes	Yes
*Hystrix brachyura*	No	Yes
*Hystrix cristata*	No	Yes
*Jaculus jaculus*	No	Yes
*Lophiomys imhausi*	No	Yes
*Mastomys coucha*	Yes	Yes
*Mastomys natalensis*	Yes	Yes
*Meriones unguiculatus*	Yes	Yes
*Mesocricetus auratus*	Yes	Yes
*Microtus agrestis*	Yes	Yes
*Microtus arvalis*	No	Yes
*Microtus fortis*	Yes	Yes
*Microtus montanus*	Yes	Yes
*Microtus ochrogaster*	Yes	Yes
*Microtus oeconomus*	Yes	Yes
*Microtus oregoni*	No	Yes
*Microtus richardsoni*	Yes	Yes
*Mus caroli*	Yes	Yes
*Mus minutoides*	Yes	Yes
*Mus musculus*	Yes	Yes
*Mus pahari*	Yes	Yes
*Mus spicilegus*	Yes	Yes
*Mus spretus*	Yes	Yes
*Muscardinus avellanarius*	No	Yes
*Myocastor coypus*	No	Yes
*Myodes glareolus*	Yes	Yes
*Nannospalax galili*	No	Yes
*Neotoma lepida*	Yes	Yes
*Ondatra zibethicus*	Yes	Yes
*Onychomys torridus*	No	Yes
*Orientallactaga bullata*	No	Yes
*Pedetes capensis*	No	Yes
*Peromyscus attwateri*	Yes	Yes
*Peromyscus aztecus*	Yes	Yes
*Peromyscus californicus*	Yes	Yes
*Peromyscus eremicus*	Yes	Yes
*Peromyscus leucopus*	Yes	Yes
*Peromyscus maniculatus*	Yes	Yes
*Peromyscus melanophrys*	Yes	Yes
*Peromyscus nudipes*	Yes	Yes
*Peromyscus polionotus*	Yes	Yes
*Phodopus roborovskii*	Yes	Yes
*Phodopus sungorus*	No	Yes
*Praomys delectorum*	Yes	Yes
*Psammomys obesus*	Yes	Yes
*Rattus norvegicus*	Yes	Yes
*Rattus rattus*	Yes	Yes
*Rhabdomys dilectus*	Yes	Yes
*Rhizomys pruinosus*	No	Yes
*Rhombomys opimus*	Yes	Yes
*Rhynchomys soricoides*	Yes	Yes
*Sigmodon hispidus*	No	Yes
*Thryonomys swinderianus*	No	Yes
*Typhlomys cinereus*	No	Yes
*Zapus hudsonius*	No	Yes

### The viral strategy targets a wide range of nonrodent mammalian species

Last, we performed a BLAST search on all available RefSeq sequences in mammals and found gRNA target sequences in the *Oxtr* gene in many more mammalian species (Table 2). This search predicted that gRNA(ΔOXTR.1) specifically targets the *Oxtr* gene in five nonrodent mammalian species and that gRNA(ΔOXTR.2) is effective in 67 nonrodent mammals. This search further indicated that gRNA(ΔOXTR.2) targets vasopressin 1b (*Avpr1b*) in 127 nonrodent mammalian species (Table 3).

**Table 2.** List of mammalian species that contain gRNA(ΔOXTR) target sequences in their *Oxtr* gene.

Species	gRNA(ΔOXTR.1)	gRNA(ΔOXTR.2)
*Acomys russatus*	No	Yes
*Ailuropoda melanoleuca*	No	Yes
*Aotus nancymaae*	No	Yes
*Apodemus sylvaticus*	No	Yes
*Arvicanthis niloticus*	Yes	No
*Arvicola amphibius*	Yes	Yes
*Balaenoptera acutorostrata scammoni*	No	Yes
*Balaenoptera musculus*	No	Yes
*Callithrix jacchus*	No	Yes
*Canis lupus dingo*	No	Yes
*Canis lupus familiaris*	No	Yes
*Cavia porcellus*	No	Yes
*Cebus imitator*	No	Yes
*Ceratotherium simum simum*	No	Yes
*Choloepus didactylus*	No	Yes
*Chrysochloris asiatica*	No	Yes
*Cricetulus griseus*	Yes	Yes
*Dipodomys ordii*	No	Yes
*Dipodomys spectabilis*	No	Yes
*Echinops telfairi*	No	Yes
*Elephantulus edwardii*	No	Yes
*Elephas maximus indicus*	No	Yes
*Eptesicus fuscus*	No	Yes
*Equus asinus*	No	Yes
*Equus caballus*	No	Yes
*Equus przewalskii*	No	Yes
*Equus quagga*	No	Yes
*Erinaceus europaeus*	No	Yes
*Fukomys damarensis*	No	Yes
*Galeopterus variegatus*	No	Yes
*Globicephala melas*	No	Yes
*Grammomys surdaster*	Yes	Yes
*Heterocephalus glaber*	No	Yes
*Hipposideros armiger*	No	Yes
*Jaculus jaculus*	No	Yes
*Lagenorhynchus obliquidens*	No	Yes
*Lemur catta*	No	Yes
*Lipotes vexillifer*	No	Yes
*Manis javanica*	No	Yes
*Manis pentadactyla*	No	Yes
*Mastomys coucha*	Yes	Yes
*Meles meles*	No	Yes
*Meriones unguiculatus*	Yes	Yes
*Mesocricetus auratus*	Yes	Yes
*Microcebus murinus*	No	Yes
*Microtus fortis*	Yes	Yes
*Microtus ochrogaster*	Yes	Yes
*Microtus oregoni*	No	Yes
*Miniopterus natalensis*	No	Yes
*Monodon monoceros*	No	Yes
*Mus caroli*	Yes	Yes
*Mus musculus*	Yes	Yes
*Mus pahari*	Yes	Yes
*Mustela erminea*	No	Yes
*Myodes glareolus*	Yes	Yes
*Myotis brandtii*	No	Yes
*Myotis davidii*	No	Yes
*Myotis lucifugus*	No	Yes
*Myotis myotis*	No	Yes
*Nannospalax galili*	No	Yes
*Neophocaena asiaeorientalis asiaeorientalis*	No	Yes
*Ochotona princeps*	No	Yes
*Onychomys torridus*	No	Yes
*Orcinus orca*	No	Yes
*Oryctolagus cuniculus*	No	Yes
*Otolemur garnettii*	No	Yes
*Peromyscus californicus insignis*	Yes	Yes
*Peromyscus leucopus*	Yes	Yes
*Peromyscus maniculatus bairdii*	Yes	Yes
*Phocoena sinus*	No	Yes
*Phodopus roborovskii*	Yes	Yes
*Pipistrellus kuhlii*	No	Yes
*Propithecus coquereli*	No	Yes
*Rattus norvegicus*	Yes	Yes
*Rattus rattus*	Yes	Yes
*Rhinolophus ferrumequinum*	No	Yes
*Rhinolophus sinicus*	No	Yes
*Saimiri boliviensis boliviensis*	No	Yes
*Sapajus apella*	No	Yes
*Talpa occidentalis*	No	Yes
*Trichechus manatus latirostris*	No	Yes
*Tupaia chinensis*	No	Yes
*Tursiops truncatus*	Yes	Yes
*Ursus americanus*	No	Yes
*Ursus arctos*	No	Yes
*Ursus maritimus*	No	Yes
*Vulpes lagopus*	No	Yes
*Vulpes vulpes*	No	Yes
		

**Table 3.** List of mammalian species that contain gRNA(ΔOXTR) target sequences in their *Avpr1b* gene.

Species	gRNA(ΔOXTR.1)	gRNA(ΔOXTR.2)
*Acinonyx jubatus*	No	Yes
*Ailuropoda melanoleuca*	No	Yes
*Aotus nancymaae*	No	Yes
*Artibeus jamaicensis*	No	Yes
*Balaenoptera acutorostrata scammoni*	No	Yes
*Balaenoptera musculus*	No	Yes
*Bison bison bison*	No	Yes
*Bos indicus*	No	Yes
*Bos indicus* × *Bos taurus*	No	Yes
*Bos mutus*	No	Yes
*Bos taurus*	No	Yes
*Bubalus bubalis*	No	Yes
*Budorcas taxicolor*	No	Yes
*Callorhinus ursinus*	No	Yes
*Camelus bactrianus*	No	Yes
*Camelus dromedarius*	No	Yes
*Camelus ferus*	No	Yes
*Capra hircus*	No	Yes
*Cebus imitator*	No	Yes
*Cercocebus atys*	No	Yes
*Cervus canadensis*	No	Yes
*Cervus elaphus*	No	Yes
*Chlorocebus sabaeus*	No	Yes
*Choloepus didactylus*	No	Yes
*Colobus angolensis palliatus*	No	Yes
*Dasypus novemcinctus*	No	Yes
*Delphinapterus leucas*	No	Yes
*Echinops telfairi*	No	Yes
*Enhydra lutris kenyoni*	No	Yes
*Eptesicus fuscus*	No	Yes
*Equus asinus*	No	Yes
*Equus caballus*	No	Yes
*Equus przewalskii*	No	Yes
*Equus quagga*	No	Yes
*Erinaceus europaeus*	No	Yes
*Eumetopias jubatus*	No	Yes
*Felis catus*	No	Yes
*Globicephala melas*	No	Yes
*Gorilla gorilla gorilla*	No	Yes
*Halichoerus grypus*	No	Yes
*Homo sapiens*	No	Yes
*Hyaena hyaena*	No	Yes
*Hylobates moloch*	No	Yes
*Lagenorhynchus obliquidens*	No	Yes
*Lemur catta*	No	Yes
*Leopardus geoffroyi*	No	Yes
*Leptonychotes weddellii*	No	Yes
*Lipotes vexillifer*	No	Yes
*Lontra canadensis*	No	Yes
*Lutra lutra*	No	Yes
*Lynx canadensis*	No	Yes
*Lynx rufus*	No	Yes
*Macaca fascicularis*	No	Yes
*Macaca mulatta*	No	Yes
*Macaca nemestrina*	No	Yes
*Macaca thibetana thibetana*	No	Yes
*Mandrillus leucophaeus*	No	Yes
*Manis pentadactyla*	No	Yes
*Meles meles*	No	Yes
*Microcebus murinus*	No	Yes
*Miniopterus natalensis*	No	Yes
*Mirounga angustirostris*	No	Yes
*Mirounga leonina*	No	Yes
*Monodon monoceros*	No	Yes
*Mustela erminea*	No	Yes
*Mustela putorius furo*	No	Yes
*Myotis brandtii*	No	Yes
*Myotis lucifugus*	No	Yes
*Myotis myotis*	No	Yes
*Nannospalax galili*	No	Yes
*Neogale vison*	No	Yes
*Neomonachus schauinslandi*	No	Yes
*Neophocaena asiaeorientalis asiaeorientalis*	No	Yes
*Nomascus leucogenys*	No	Yes
*Odobenus rosmarus divergens*	No	Yes
*Odocoileus virginianus texanus*	No	Yes
*Orcinus orca*	No	Yes
*Oryx dammah*	No	Yes
*Ovis aries*	No	Yes
*Pan paniscus*	No	Yes
*Pan troglodytes*	No	Yes
*Panthera leo*	No	Yes
*Panthera pardus*	No	Yes
*Panthera tigris*	No	Yes
*Panthera uncia*	No	Yes
*Papio anubis*	No	Yes
*Phacochoerus africanus*	No	Yes
*Phoca vitulina*	No	Yes
*Phocoena sinus*	No	Yes
*Phyllostomus discolor*	No	Yes
*Phyllostomus hastatus*	No	Yes
*Physeter catodon*	No	Yes
*Piliocolobus tephrosceles*	No	Yes
*Pipistrellus kuhlii*	No	Yes
*Pongo abelii*	No	Yes
*Prionailurus bengalensis*	No	Yes
*Prionailurus viverrinus*	No	Yes
*Propithecus coquereli*	No	Yes
*Pteropus alecto*	No	Yes
*Pteropus giganteus*	No	Yes
*Pteropus vampyrus*	No	Yes
*Puma concolor*	No	Yes
*Puma yagouaroundi*	No	Yes
*Rhinolophus ferrumequinum*	No	Yes
*Rhinolophus sinicus*	No	Yes
*Rhinopithecus bieti*	No	Yes
*Rhinopithecus roxellana*	No	Yes
*Rousettus aegyptiacus*	No	Yes
*Saimiri boliviensis boliviensis*	No	Yes
*Sapajus apella*	No	Yes
*Sturnira hondurensis*	No	Yes
*Suncus etruscus*	No	Yes
*Sus scrofa*	No	Yes
*Talpa occidentalis*	No	Yes
*Theropithecus gelada*	No	Yes
*Trachypithecus francoisi*	No	Yes
*Tupaia chinensis*	No	Yes
*Tursiops truncatus*	No	Yes
*Ursus americanus*	No	Yes
*Ursus arctos*	No	Yes
*Ursus arctos horribilis*	No	Yes
*Ursus maritimus*	No	Yes
*Vicugna pacos*	No	Yes
*Zalophus californianus*	No	Yes

## DISCUSSION

We here developed a specific, highly efficient tool to reduce functional OXTR densities in multiple rodent species used in sociobehavioral studies. We targeted the *Oxtr* coding sequence in five different brain regions across six rodent species and reduced OXTR levels in all cases. Injection of AAV-ΔOXTR did not result in gross neurotoxicity, as demonstrated by unaffected NeuN expression, nor did it reduce the protein density of the most probable off-target gene product, AVPR1A. Thereby, we validated this method for use in comparative OXT research.

Our tool represents a major advancement over other techniques that are used to genetically manipulate OXTR levels. It is more effective than short hairpin RNA (shRNA)–mediated knockdown (*36*) and more versatile and selective than systemic knockouts. The ability to reduce OXTR density in multiple species is of great value to OXT research, which has a strong comparative tradition. One of the guiding hypotheses that have emerged from comparative OXT research is that species-specific *Oxtr* expression patterns influence the regulation of social behaviors (*29*, *33*, *47*, *48*) and that OXTR in different circuits can exert different effects on behavior (*49*). For example, socially monogamous species of voles have higher densities of OXTR in the NAc than do nonmonogamous species, and manipulating OXTR density in that region affects pair bonding behaviors (*20*). OXTR signaling in the NAc is critical for pair bond formation and maintenance (*38*), by modulating the salience of social stimuli (*50*), while genetically mediated variation in OXTR in the NAc modulates pair bonding and the effects of early-life experience on pair bonding (*34*, *35*, *51*). In addition, OXTR levels tend to be high in brain areas that associate with the primary sensory modality of a species (*52*). In rodents, the olfactory system is densely populated with OXTR, while in primates the visual system has high levels of OXTR. Therefore, variation in OXTR distribution is thought to underlie differences in social strategies and contribute to diversity in social behaviors (*53*, *54*). In theory, future comparative genome editing strategies could facilitate testing of this hypothesis by allowing direct comparison of OXTR function across species.

A limitation of our strategy is that while it targets many species, it does not target all species. Part of this limitation stems from the evolvability of the target gene, as it will be easier to design comparative gene editing strategies to target conserved genes, rather than for genes of which many variants exist. For genes with abundant sequence diversity, only the use of multiple gRNAs can ensure the targeting of all gene variants. However, the relative ease with which gRNAs can be multiplexed in AAV vectors should make it possible to target a wide variety of species with one single strategy. It is also important to consider that while two-thirds of the mutated gene products are likely to be nonfunctional because of frameshift mutations, one-third of mutated gene products might retain some functionality (e.g., receptor dimerization) (*55*). While our tool induces a near-complete loss of a specific functionality of the OXT receptor (i.e., ligand binding), we cannot exclude the possibility that a small part of the mutated gene products retains some form of residual activity. However, the tool is easily adapted to target other protein domains for functional characterization. Perhaps the greatest challenge to the efficient applicability of comparative genome editing is the design of gRNAs that target the widest range of species with preservation of specificity and efficiency. To simplify the design of comparative gene editing, gRNA-specific computational tools will have to be developed.

This work demonstrates the feasibility of designing genetic tools that are functional in multiple species. Although our tool was designed to target rodent *Oxtr* coding sequences, it is predicted to target many nonrodent mammalian species as well. This shows that the design of genetic tools that work in multiple species is not limited to a single order but can be designed to target a much wider range of species. One of the main advantages of widely used neuroscience techniques, like chemo- and optogenetics, is that they can be used in a wide array of species (*19*, *56*–*58*). This not only has given tremendous insight into the functioning of neural circuits but also has direct translational value, precisely because these techniques function in many species and thus allow the elucidation of general and species-specific principles of gene-brain-behavior relationships. Therefore, we believe that the comparative design of genetic tools will greatly enhance the translatability of future genetic techniques, both when used in research as well as in the clinic. In sum, we hope that this work will encourage the application of genetic dissection in comparative neuroscience and thereby advance our understanding of the general and species-specific principles of neural circuit functioning.

## MATERIALS AND METHODS

### Design and synthesis of viral vectors

*Oxtr* coding sequences of *M. auratus*, *M. ochrogaster*, *M. musculus*, *and R. norvegicus* were aligned with the ClustalW algorithm in the R/Bioconductor msa package to identify conserved regions (*40*). Within conserved regions, possible gRNA sequences were identified using Benchling. Three candidate sequences were selected on the basis of predicted efficiency and off-target effects. A control gRNA was designed to target the bacterial *lacZ* gene. gRNA sequences cloned into pAAV-U6-gRNA-CMV-eGFP were the following: gRNA(ΔOXTR.1), 5′-GGTGCTTCATGAAAAAGAAG-3′; gRNA(ΔOXTR.2), 5′-GTGATGTCCCACAGCAGCTG-3′; gRNA(ΔOXTR.3), 5′-GCCCGACCTGCTGTGTCGTC-3′; and gRNA(CTRL), 5′-GTGAGCGAGTAACAACCCGT-3′. Oligos were cloned into pAAV-U6-gRNA-CMV-eGFP (Addgene, plasmid #85451, gift of H. Lei) after plasmid digestion with Sap I. The first batch of viral particles was synthesized using pAAV-U6-gRNA-CMV-eGFP, pAAV-RSV-spCas9 (Addgene, plasmid #85450, gifted by H. Lei), pAAV9-SPAKFA (Penn Vector Core, PA, USA), and pAAV/Ad (American Type Culture Collection, VA, USA). AAV9 particles were produced in human embryonic kidney (HEK) 293T cells, purified with AVB-affinity chromatography (*59*), and concentrated by centrifugal filtration (Amicon Ultra-4, Fisher Scientific, NH, USA), after which viral titer was determined using quantitative PCR targeting the inverted terminal repeats (*60*). Five viruses were generated: AAV9-U6- gRNA(ΔOXTR.1)-CMV-eGFP, AAV9-U6-gRNA(ΔOXTR.2)-CMV-eGFP, AAV9-U6-gRNA(ΔOXTR.3)-CMV-eGFP, AAV9-U6-gRNA(CTRL)-CMV-eGFP, and AAV9-RSV-spCas9. AAV-gRNA and AAV-Cas9 vectors were diluted to 3.0 × 10^10^ and 1.5 × 10^10^ genomic copies/μl, respectively, and mixed in a 1:1 ratio. In initial experiments in the prairie vole, AAV-gRNA(ΔOXTR.3) showed markedly lower efficiency and was not tested further. A second batch of AAV9-viruses was generated by VectorBuilder (Chicago, IL, USA): AAV9-U6-gRNA(ΔOXTR.1)-CMV-eGFP (2.0 × 10^10^ genomic copies/μl), AAV9-U6-gRNA(ΔOXTR.2)-CMV-eGFP, AAV9-U6-gRNA(CTRL)-CMV-eGFP (2.0 × 10^10^ genomic copies/μl), and AAV9-RSV-spCas9 (1.5 × 10^10^ genomic copies/μl). Both batches of the virus showed similar efficacy, and data from both batches have been pooled.

### Animals

All experiments were performed following the guidelines and approved by the respective Institutional Animal Care and Use Committees (Emory University, Georgia State University, and University of California, Davis). The following animals were used in this study: California deer mice (B.C.T., University of California, Davis: 3 males and 3 females), golden hamsters (H.E.A., Georgia State University: 3 males and 3 females), house mice (L.J.Y., Emory University: 7 males), Norway rat (L.J.Y., Emory University: 10 males), prairie vole (L.J.Y., Emory University: 16 males and 16 females), and spiny mice (A.M.K., Emory University: 3 males and 9 females). All animals were sexually naïve adults and housed in standard laboratory conditions with ad libitum water and food provided.

### Intracranial surgeries

For all species, anesthesia was induced by exposure to a 2 to 4% isoflurane/oxygen mix and maintained at 1 to 3%. Three daily doses of meloxicam or carprofen (2 to 5 mg/kg, depending on species) were administered after surgery. Using a stereotaxic apparatus, animals were unilaterally injected with a 1:1 mix of AAV9-RSV-Cas9 and AAV9-gRNA(ΔOXTR), while the contralateral side received a 1:1 mix of AAV9-RSV-Cas9 and AAV9-gRNA(CTRL). Stereotaxic coordinates and injected volumes are summarized in Table 4.

**Table 4.** Stereotaxic coordinates and injected volume. AP, anterior-posterior; ML, medial-lateral; DV, dorsal-ventral; EC, endopiriform cortex; VP, ventral pallidum; LS, lateral septum; CeA, central amygdala.

Common name	Area	AP	ML	DV	Angle	Volume (nl)
Spiny mouse	NAc	+2.9	±1	−5	0	300
Golden hamster	EC	+3.6	±3.2	−6	0	200
House mouse	VMH	−1.22	±2.0	−5.3	0	300
Prairie vole	NAc	+1.7	±2.1	−4.7	10	300
Prairie vole	VP	+0.2	±1.8	−5.1	10	300
California deer mouse	LS	−0.36	±0.7	−3.5	0	300
Norway rat	CeA	−1.8	±4.2	−8	0	300

### T7 endonuclease I assay

eGFP-infected tissue was collected from fresh-frozen brain sections, and DNA was isolated using the Blood & Tissue DNA kit (Qiagen, Germany). Fragments surrounding the gRNA target sides were PCR-amplified using Q5-polymerase (New England Biolabs, MA, USA) and the primer set 5′-AGCAGTCAAAAACACCGTCC-3′ (forward) and 5′-GACACCTGGACAACTCATCGG-3′ (reverse) under these cycling conditions: 98°C for 2 min, 34 cycles of 98°C for 15 s, 63°C for 15 s, and 72°C for 30 s, and 2 min of final elongation. Next, PCR fragments were used for the T7 endonuclease I assay according to the manufacturer’s instructions (Integrated DNA Technologies, IA, USA).

### OXTR and AVPR1A autoradiography

Fresh-frozen brains were sectioned on a cryostat (Epredia Cryostar NX-70, Thermo Fisher Scientific, MA, USA) at 20 μm, mounted on Superfrost Plus slides (Fisher Scientific), and stored at −80°C until use. Autoradiography was performed as previously described (*41*). Briefly, slides were thawed and fixed for 2 min in 0.1% paraformaldehyde in phosphate-buffered saline (PBS) for 2 min, washed in 50 mM tris in PBS (pH 7.4, 2 × 10 min), and incubated in 50 mM tris buffer, supplemented with 0.1% bovine serum albumin and 50 pM I^125^-OVTA (2200 Ci/mmol, ornithine vasotocin analog, #NEX254010UC, PerkinElmer, MA, USA) or 50 pM I^125^-AVP (2200 Ci/mmol, linear arginine vasopressin, #NEX310010UC, PerkinElmer) at room temperature (RT) for 1 hour. Unbound ligand was removed by washing in 50 mM tris with 0.2% MgCl_2_ at 4°C (4 × 5 min) and 30 min at RT. Slides were dipped in Milli-Q, dried, and placed in a cassette with BioMax MR film (Sigma-Aldrich, MO, USA). After 7 days, films were developed and imaged using an MCID core system (Interfocus Co., UK). Mean gray values of viral-targeted regions, corrected for background, were determined in ImageJ. I^125^-activity (disintegrations per minute) was calculated using an I^125^-standard and taken as a proxy for OXTR or AVPR1A density. Differences in OXTR or AVPR1A density were determined by comparing protein density levels in AAV-ΔOXTR–injected regions to protein density levels in contralateral AAV-CTRL–injected regions.

### Immunohistochemistry

Animals were deeply anesthetized and transcardially perfused with PBS, followed by PBS supplemented with 4% paraformaldehyde. Brains were sectioned on a cryostat (Cryostar NX-70) at 40 μm and stored in cryoprotectant buffer at −20°C until use. Sections were thawed, washed in PBS, and blocked and permeabilized in PBS supplemented with 0.1% Tween (PBST) and 5% normal donkey serum for 1 hour at RT. Next, sections were incubated in PBST supplemented with 0.5% rabbit anti-NeuN (1:1000, AB104225, Abcam, UK) at 4°C overnight. After PBS washes, sections were incubated in anti-rabbit Alexa Fluor 568 antibodies (1:500, Molecular Probes, OR, USA) for 1 hour. Sections were mounted and coverslipped in Fluoromount-G containing 4′,6-diamidino-2-phenylindole (DAPI; Thermo Fisher Scientific). Z-stacks with ×60 magnification of eGFP-injected regions and the corresponding sham-injected contralateral region were imaged using a Keyence microscope. Z-stacks were projected with maximal intensity, and NeuN-positive cells were manually counted in ImageJ by a blinded observer.

### In situ hybridization

Fresh-frozen brains (*N* = 2 × 2 hemispheres) were sectioned on a cryostat (Cryostar NX-70) at 20 μm, mounted on Superfrost Plus slides (Fisher Scientific), and stored at −80°C until use. Slides were thawed, and RNAscope in situ hybridization was done according to the manufacturer’s protocol (#320293, ACD Inc., MN, USA). Briefly, slides were pretreated with Protease (#320842, ACD Inc.) and incubated with spCas9 probes (#519411, ACD Inc.) for 2 hours at 40°C. Sections were washed, and signal amplification was performed using the kit’s reagents. Last, sections were coverslipped in Fluoromount-G containing DAPI (Thermo Fisher Scientific). The 20× images were made on a Keyence BZ-X700 microscope (Keyence, Japan).

### Statistical analyses

All statistical analyses were performed in RStudio, using paired *t* tests (Figs. 1, E and F, 2B, and 3F and fig. S1B), with α = 0.05.

### BLAST

We used the blastn algorithm in the NCBI BLAST+ software suite to search for perfect alignment of the target RNA sequences plus the permissive PAM sequence (5′-NGG-3′) to rodent (taxid 9989) and mammalian (taxid 40674) genomes to identify species in which our tool is predicted to be functional.

## References

[1] R. J. V. Roberts, S. Pop, L. L. Prieto-Godino, Evolution of central neural circuits: State of the art and perspectives. Nat. Rev. Neurosci. 23, 725–743 (2022). 36289403 10.1038/s41583-022-00644-y

[2] A. S. Mathuru, F. Libersat, A. Vyas, S. Teseo, Why behavioral neuroscience still needs diversity? A curious case of a persistent need. Neurosci. Biobehav. Rev. 116, 130–141 (2020). 32565172 10.1016/j.neubiorev.2020.06.021

[3] A. J. Boender, L. Bontempi, L. Nava, Y. Pelloux, R. Tonini, Striatal astrocytes shape behavioral flexibility via regulation of the glutamate transporter EAAT2. Biol. Psychiatry 89, 1045–1057 (2021). 33516457 10.1016/j.biopsych.2020.11.015

[4] A. J. Boender, M. A. van Gestel, K. M. Garner, M. C. Luijendijk, R. A. Adan, The obesity-associated gene Negr1 regulates aspects of energy balance in rat hypothalamic areas. Physiol. Rep. 2, e12083 (2014). 25077509 10.14814/phy2.12083 PMC4187548

[5] J. Wahis, A. Baudon, F. Althammer, D. Kerspern, S. Goyon, D. Hagiwara, A. Lefevre, L. Barteczko, B. Boury-Jamot, B. Bellanger, M. Abatis, M. da Silva Gouveia, D. Benusiglio, M. Eliava, A. Rozov, I. Weinsanto, H. S. Knobloch-Bollmann, M. K. Kirchner, R. K. Roy, H. Wang, M. Pertin, P. Inquimbert, C. Pitzer, J. Siemens, Y. Goumon, B. Boutrel, C. M. Lamy, I. Decosterd, J. Y. Chatton, N. Rouach, W. S. Young, J. E. Stern, P. Poisbeau, R. Stoop, P. Darbon, V. Grinevich, A. Charlet, Astrocytes mediate the effect of oxytocin in the central amygdala on neuronal activity and affective states in rodents. Nat. Neurosci. 24, 529–541 (2021). 33589833 10.1038/s41593-021-00800-0

[6] J. LeDoux, *Anxious: Using the Brain to Understand and Treat Fear and Anxiety* (Penguin Publishing Group, 2015).

[7] A. Rhie, S. A. McCarthy, O. Fedrigo, J. Damas, G. Formenti, S. Koren, M. Uliano-Silva, W. Chow, A. Fungtammasan, J. Kim, C. Lee, B. J. Ko, M. Chaisson, G. L. Gedman, L. J. Cantin, F. Thibaud-Nissen, L. Haggerty, I. Bista, M. Smith, B. Haase, J. Mountcastle, S. Winkler, S. Paez, J. Howard, S. C. Vernes, T. M. Lama, F. Grutzner, W. C. Warren, C. N. Balakrishnan, D. Burt, J. M. George, M. T. Biegler, D. Iorns, A. Digby, D. Eason, B. Robertson, T. Edwards, M. Wilkinson, G. Turner, A. Meyer, A. F. Kautt, P. Franchini, H. W. Detrich III, H. Svardal, M. Wagner, G. J. P. Naylor, M. Pippel, M. Malinsky, M. Mooney, M. Simbirsky, B. T. Hannigan, T. Pesout, M. Houck, A. Misuraca, S. B. Kingan, R. Hall, Z. Kronenberg, I. Sović, C. Dunn, Z. Ning, A. Hastie, J. Lee, S. Selvaraj, R. E. Green, N. H. Putnam, I. Gut, J. Ghurye, E. Garrison, Y. Sims, J. Collins, S. Pelan, J. Torrance, A. Tracey, J. Wood, R. E. Dagnew, D. Guan, S. E. London, D. F. Clayton, C. V. Mello, S. R. Friedrich, P. V. Lovell, E. Osipova, F. O. al-Ajli, S. Secomandi, H. Kim, C. Theofanopoulou, M. Hiller, Y. Zhou, R. S. Harris, K. D. Makova, P. Medvedev, J. Hoffman, P. Masterson, K. Clark, F. Martin, K. Howe, P. Flicek, B. P. Walenz, W. Kwak, H. Clawson, M. Diekhans, L. Nassar, B. Paten, R. H. S. Kraus, A. J. Crawford, M. T. P. Gilbert, G. Zhang, B. Venkatesh, R. W. Murphy, K. P. Koepfli, B. Shapiro, W. E. Johnson, F. di Palma, T. Marques-Bonet, E. C. Teeling, T. Warnow, J. M. Graves, O. A. Ryder, D. Haussler, S. J. O’Brien, J. Korlach, H. A. Lewin, K. Howe, E. W. Myers, R. Durbin, A. M. Phillippy, E. D. Jarvis, Towards complete and error-free genome assemblies of all vertebrate species. Nature 592, 737–746 (2021). 33911273 10.1038/s41586-021-03451-0 PMC8081667

[8] M. Jinek, K. Chylinski, I. Fonfara, M. Hauer, J. A. Doudna, E. Charpentier, A programmable dual-RNA-guided DNA endonuclease in adaptive bacterial immunity. Science 337, 816–821 (2012). 22745249 10.1126/science.1225829 PMC6286148

[9] L. Cong, F. A. Ran, D. Cox, S. Lin, R. Barretto, N. Habib, P. D. Hsu, X. Wu, W. Jiang, L. A. Marraffini, F. Zhang, Multiplex genome engineering using CRISPR/Cas systems. Science 339, 819–823 (2013). 23287718 10.1126/science.1231143 PMC3795411

[10] L. Swiech, M. Heidenreich, A. Banerjee, N. Habib, Y. Li, J. Trombetta, M. Sur, F. Zhang, In vivo interrogation of gene function in the mammalian brain using CRISPR-Cas9. Nat. Biotechnol. 33, 102–106 (2015). 25326897 10.1038/nbt.3055 PMC4492112

[11] A. J. Boender, L. J. Young, Oxytocin, vasopressin and social behavior in the age of genome editing: A comparative perspective. Horm. Behav. 124, 104780 (2020). 32544402 10.1016/j.yhbeh.2020.104780 PMC7486992

[12] K. Horie, K. Inoue, K. Nishimori, L. J. Young, Investigation of *Oxtr*-expressing neurons projecting to nucleus accumbens using *Oxtr-ires-Cre* knock-in prairie voles (*Microtus ochrogaster*). Neuroscience 448, 312–324 (2020). 33092784 10.1016/j.neuroscience.2020.08.023 PMC7643779

[13] K. Horie, K. Inoue, S. Suzuki, S. Adachi, S. Yada, T. Hirayama, S. Hidema, L. J. Young, K. Nishimori, Oxytocin receptor knockout prairie voles generated by CRISPR/Cas9 editing show reduced preference for social novelty and exaggerated repetitive behaviors. Horm. Behav. 111, 60–69 (2019). 30713102 10.1016/j.yhbeh.2018.10.011 PMC6506400

[14] S. Yokoi, K. Naruse, Y. Kamei, S. Ansai, M. Kinoshita, M. Mito, S. Iwasaki, S. Inoue, T. Okuyama, S. Nakagawa, L. J. Young, H. Takeuchi, Sexually dimorphic role of oxytocin in medaka mate choice. Proc. Natl. Acad. Sci. U.S.A. 117, 4802–4808 (2020). 32071244 10.1073/pnas.1921446117 PMC7060708

[15] J. H. Taylor, J. C. Walton, K. E. McCann, A. Norvelle, Q. Liu, J. W. Vander Velden, J. M. Borland, M. Hart, C. Jin, K. L. Huhman, D. N. Cox, H. E. Albers, CRISPR-Cas9 editing of the arginine-vasopressin V1a receptor produces paradoxical changes in social behavior in Syrian hamsters. Proc. Natl. Acad. Sci. U.S.A. 119, e2121037119 (2022). 35512092 10.1073/pnas.2121037119 PMC9171636

[16] Z. A. Grieb, E. A. Cross, H. E. Albers, Alpha-melanocyte-stimulating hormone (αMSH) modulates the rewarding properties of social interactions in an oxytocin receptor-dependent manner in Syrian hamsters (Mesocricetus Auratus). Physiol. Behav. 252, 113828 (2022). 35500727 10.1016/j.physbeh.2022.113828 PMC10858742

[17] A. V. Williams, N. Duque-Wilckens, S. Ramos-Maciel, K. L. Campi, S. K. Bhela, C. K. Xu, K. Jackson, B. Chini, P. A. Pesavento, B. C. Trainor, Social approach and social vigilance are differentially regulated by oxytocin receptors in the nucleus accumbens. Neuropsychopharmacology 45, 1423–1430 (2020). 32198453 10.1038/s41386-020-0657-4 PMC7360746

[18] N. Duque-Wilckens, L. Y. Torres, S. Yokoyama, V. A. Minie, A. M. Tran, S. P. Petkova, R. Hao, S. Ramos-Maciel, R. A. Rios, K. Jackson, F. J. Flores-Ramirez, I. Garcia-Carachure, P. A. Pesavento, S. D. Iñiguez, V. Grinevich, B. C. Trainor, Extrahypothalamic oxytocin neurons drive stress-induced social vigilance and avoidance. Proc. Natl. Acad. Sci. U.S.A. 117, 26406–26413 (2020). 33020267 10.1073/pnas.2011890117 PMC7585015

[19] Z. He, L. Zhang, W. Hou, X. Zhang, L. J. Young, L. Li, L. Liu, H. Ma, Y. Xun, Z. Lv, Y. Li, R. Jia, J. Li, F. Tai, Paraventricular nucleus oxytocin subsystems promote active paternal behaviors in mandarin voles. J. Neurosci. 41, 6699–6713 (2021). 34226275 10.1523/JNEUROSCI.2864-20.2021 PMC8336703

[20] H. E. Ross, S. M. Freeman, L. L. Spiegel, X. Ren, E. F. Terwilliger, L. J. Young, Variation in oxytocin receptor density in the nucleus accumbens has differential effects on affiliative behaviors in monogamous and polygamous voles. J. Neurosci. 29, 1312–1318 (2009). 19193878 10.1523/JNEUROSCI.5039-08.2009 PMC2768419

[21] V. Ferretti, F. Maltese, G. Contarini, M. Nigro, A. Bonavia, H. Huang, V. Gigliucci, G. Morelli, D. Scheggia, F. Managò, G. Castellani, A. Lefevre, L. Cancedda, B. Chini, V. Grinevich, F. Papaleo, Oxytocin signaling in the central amygdala modulates emotion discrimination in mice. Curr. Biol. 29, 1938–1953.e6 (2019). 31178317 10.1016/j.cub.2019.04.070

[22] Y. Tang, D. Benusiglio, A. Lefevre, L. Hilfiger, F. Althammer, A. Bludau, D. Hagiwara, A. Baudon, P. Darbon, J. Schimmer, M. K. Kirchner, R. K. Roy, S. Wang, M. Eliava, S. Wagner, M. Oberhuber, K. K. Conzelmann, M. Schwarz, J. E. Stern, G. Leng, I. D. Neumann, A. Charlet, V. Grinevich, Social touch promotes interfemale communication via activation of parvocellular oxytocin neurons. Nat. Neurosci. 23, 1125–1137 (2020). 32719563 10.1038/s41593-020-0674-y

[23] S. Yokoi, T. Okuyama, Y. Kamei, K. Naruse, Y. Taniguchi, S. Ansai, M. Kinoshita, L. J. Young, N. Takemori, T. Kubo, H. Takeuchi, An essential role of the arginine vasotocin system in mate-guarding behaviors in triadic relationships of medaka fish (*Oryzias latipes*). PLOS Genet. 11, e1005009 (2015). 25719383 10.1371/journal.pgen.1005009 PMC4342251

[24] J. C. Burkhart, S. Gupta, N. Borrego, S. R. Heilbronner, C. Packer, Oxytocin promotes social proximity and decreases vigilance in groups of African lions. iScience 25, 104049 (2022). 35496998 10.1016/j.isci.2022.104049 PMC9042884

[25] C. Crockford, T. Deschner, R. M. Wittig, The role of oxytocin in social buffering: What do primate studies add? Curr. Top. Behav. Neurosci. 35, 155–173 (2018). 28864973 10.1007/7854_2017_12

[26] J. L. Garrison, E. Z. Macosko, S. Bernstein, N. Pokala, D. R. Albrecht, C. I. Bargmann, Oxytocin/vasopressin-related peptides have an ancient role in reproductive behavior. Science 338, 540–543 (2012). 23112335 10.1126/science.1226201 PMC3597094

[27] Z. Triki, K. Daughters, C. K. W. De Dreu, Oxytocin has ‘tend-and-defend’ functionality in group conflict across social vertebrates. Philos. Trans. R. Soc. Lond. B Biol. Sci. 377, 20210137 (2022). 35369742 10.1098/rstb.2021.0137 PMC8977669

[28] N. Rigney, G. J. de Vries, A. Petrulis, L. J. Young, Oxytocin, vasopressin, and social behavior: From neural circuits to clinical opportunities. Endocrinology 163, bqac111 (2022). 35863332 10.1210/endocr/bqac111 PMC9337272

[29] R. C. Froemke, L. J. Young, Oxytocin, neural plasticity, and social behavior. Annu. Rev. Neurosci. 44, 359–381 (2021). 33823654 10.1146/annurev-neuro-102320-102847 PMC8604207

[30] C. L. Ford, L. J. Young, Refining oxytocin therapy for autism: Context is key. Nat. Rev. Neurol. 18, 67–68 (2022). 34880473 10.1038/s41582-021-00602-9 PMC8816821

[31] C. L. Ford, L. J. Young, Translational opportunities for circuit-based social neuroscience: Advancing 21st century psychiatry. Curr. Opin. Neurobiol. 68, 1–8 (2021). 33260106 10.1016/j.conb.2020.11.007 PMC8160019

[32] L. J. Young, C. E. Barrett, Neuroscience. Can oxytocin treat autism? Science 347, 825–826 (2015). 25700501 10.1126/science.aaa8120 PMC4362686

[33] Z. R. Donaldson, L. J. Young, Oxytocin, vasopressin, and the neurogenetics of sociality. Science 322, 900–904 (2008). 18988842 10.1126/science.1158668

[34] L. B. King, H. Walum, K. Inoue, N. W. Eyrich, L. J. Young, Variation in the oxytocin receptor gene predicts brain region-specific expression and social attachment. Biol. Psychiatry 80, 160–169 (2016). 26893121 10.1016/j.biopsych.2015.12.008 PMC4909578

[35] C. E. Barrett, S. E. Arambula, L. J. Young, The oxytocin system promotes resilience to the effects of neonatal isolation on adult social attachment in female prairie voles. Transl. Psychiatry 5, e606 (2015). 26196439 10.1038/tp.2015.73 PMC5068726

[36] A. C. Keebaugh, C. E. Barrett, J. L. Laprairie, J. J. Jenkins, L. J. Young, RNAi knockdown of oxytocin receptor in the nucleus accumbens inhibits social attachment and parental care in monogamous female prairie voles. Soc. Neurosci. 10, 561–570 (2015). 25874849 10.1080/17470919.2015.1040893 PMC4618772

[37] Y. Takayanagi, M. Yoshida, I. F. Bielsky, H. E. Ross, M. Kawamata, T. Onaka, T. Yanagisawa, T. Kimura, M. M. Matzuk, L. J. Young, K. Nishimori, Pervasive social deficits, but normal parturition, in oxytocin receptor-deficient mice. Proc. Natl. Acad. Sci. U.S.A. 102, 16096–16101 (2005). 16249339 10.1073/pnas.0505312102 PMC1276060

[38] O. J. Bosch, J. Dabrowska, M. E. Modi, Z. V. Johnson, A. C. Keebaugh, C. E. Barrett, T. H. Ahern, J. D. Guo, V. Grinevich, D. G. Rainnie, I. D. Neumann, L. J. Young, Oxytocin in the nucleus accumbens shell reverses CRFR2-evoked passive stress-coping after partner loss in monogamous male prairie voles. Psychoneuroendocrinology 64, 66–78 (2016). 26615473 10.1016/j.psyneuen.2015.11.011 PMC4698175

[39] A. M. Borie, S. Agezo, P. Lunsford, A. J. Boender, J. D. Guo, H. Zhu, G. J. Berman, L. J. Young, R. C. Liu, Social experience alters oxytocinergic modulation in the nucleus accumbens of female prairie voles. Curr. Biol. 32, 1026–1037.e4 (2022). 35108521 10.1016/j.cub.2022.01.014 PMC8930613

[40] U. Bodenhofer, E. Bonatesta, C. Horejs-Kainrath, S. Hochreiter, msa: An R package for multiple sequence alignment. Bioinformatics 31, 3997–3999 (2015). 26315911 10.1093/bioinformatics/btv494

[41] K. Inoue, C. L. Ford, K. Horie, L. J. Young, Oxytocin receptors are widely distributed in the prairie vole (*Microtus ochrogaster*) brain: Relation to social behavior, genetic polymorphisms, and the dopamine system. J. Comp. Neurol. 530, 2881–2900 (2022). 35763609 10.1002/cne.25382 PMC9474670

[42] C. Theofanopoulou, G. Gedman, J. A. Cahill, C. Boeckx, E. D. Jarvis, Universal nomenclature for oxytocin-vasotocin ligand and receptor families. Nature 592, 747–755 (2021). 33911268 10.1038/s41586-020-03040-7 PMC8081664

[43] J. H. Taylor, K. E. McCann, A. P. Ross, H. E. Albers, Binding affinities of oxytocin, vasopressin and Manning compound at oxytocin and V1a receptors in male Syrian hamster brains. J. Neuroendocrinol. 32, e12882 (2020). 32662552 10.1111/jne.12882 PMC7485222

[44] J. M. Powell, K. Inoue, K. J. Wallace, A. W. Seifert, L. J. Young, A. M. Kelly, Distribution of vasopressin 1a and oxytocin receptor protein and mRNA in the basal forebrain and midbrain of the spiny mouse (*Acomys cahirinus*). Brain Struct. Funct. 228, 413 (2023). 36271259 10.1007/s00429-022-02581-z PMC9974677

[45] K. T. Newmaster, Z. T. Nolan, U. Chon, D. J. Vanselow, A. R. Weit, M. Tabbaa, S. Hidema, K. Nishimori, E. A. D. Hammock, Y. Kim, Quantitative cellular-resolution map of the oxytocin receptor in postnatally developing mouse brains. Nat. Commun. 11, 1885 (2020). 32313029 10.1038/s41467-020-15659-1 PMC7171089

[46] S. F. Altschul, W. Gish, W. Miller, E. W. Myers, D. J. Lipman, Basic local alignment search tool. J. Mol. Biol. 215, 403–410 (1990). 2231712 10.1016/S0022-2836(05)80360-2

[47] L. J. Young, Z. Wang, The neurobiology of pair bonding. Nat. Neurosci. 7, 1048–1054 (2004). 15452576 10.1038/nn1327

[48] H. Walum, L. J. Young, The neural mechanisms and circuitry of the pair bond. Nat. Rev. Neurosci. 19, 643–654 (2018). 30301953 10.1038/s41583-018-0072-6 PMC6283620

[49] M. Q. Steinman, N. Duque-Wilckens, B. C. Trainor, Complementary neural circuits for divergent effects of oxytocin: Social approach versus social anxiety. Biol. Psychiatry 85, 792–801 (2019). 30503164 10.1016/j.biopsych.2018.10.008 PMC6709863

[50] Z. V. Johnson, H. Walum, Y. Xiao, P. C. Riefkohl, L. J. Young, Oxytocin receptors modulate a social salience neural network in male prairie voles. Horm. Behav. 87, 16–24 (2017). 27793769 10.1016/j.yhbeh.2016.10.009 PMC5207344

[51] T. H. Ahern, S. Olsen, R. Tudino, A. K. Beery, Natural variation in the oxytocin receptor gene and rearing interact to influence reproductive and nonreproductive social behavior and receptor binding. Psychoneuroendocrinology 128, 105209 (2021). 33839431 10.1016/j.psyneuen.2021.105209 PMC8131238

[52] S. M. Freeman, L. J. Young, Comparative perspectives on oxytocin and vasopressin receptor research in rodents and primates: Translational implications. J. Neuroendocrinol. 28, (2016). 10.1111/jne.12382 PMC4886472 26940141

[53] Z. V. Johnson, L. J. Young, Oxytocin and vasopressin neural networks: Implications for social behavioral diversity and translational neuroscience. Neurosci. Biobehav. Rev. 76, 87–98 (2017). 28434591 10.1016/j.neubiorev.2017.01.034 PMC5407410

[54] Z. V. Johnson, L. J. Young, Evolutionary diversity as a catalyst for biological discovery. Integr. Zool. 13, 616–633 (2018). 29851228 10.1111/1749-4877.12339 PMC6265060

[55] B. Jurek, L. Denk, N. Schäfer, M. S. Salehi, S. Pandamooz, S. Haerteis, Oxytocin accelerates tight junction formation and impairs cellular migration in 3D spheroids: Evidence from Gapmer-induced exon skipping. Front. Cell. Neurosci. 16, 1000538 (2022). 36263085 10.3389/fncel.2022.1000538 PMC9574052

[56] E. A. Amadei, Z. V. Johnson, Y. Jun Kwon, A. C. Shpiner, V. Saravanan, W. D. Mays, S. J. Ryan, H. Walum, D. G. Rainnie, L. J. Young, R. C. Liu, Dynamic corticostriatal activity biases social bonding in monogamous female prairie voles. Nature 546, 297–301 (2017). 28562592 10.1038/nature22381 PMC5499998

[57] J. Singh Alvarado, J. Goffinet, V. Michael, W. Liberti III, J. Hatfield, T. Gardner, J. Pearson, R. Mooney, Neural dynamics underlying birdsong practice and performance. Nature 599, 635–639 (2021). 34671166 10.1038/s41586-021-04004-1 PMC9118926

[58] T. Oti, K. Satoh, D. Uta, J. Nagafuchi, S. Tateishi, R. Ueda, K. Takanami, L. J. Young, A. Galione, J. F. Morris, T. Sakamoto, H. Sakamoto, Oxytocin influences male sexual activity via non-synaptic axonal release in the spinal cord. Curr. Biol. 31, 103–114.e5 (2021). 33125871 10.1016/j.cub.2020.09.089 PMC7855431

[59] Q. Wang, M. Lock, A. J. Prongay, M. R. Alvira, B. Petkov, J. M. Wilson, Identification of an adeno-associated virus binding epitope for AVB sepharose affinity resin. Mol. Ther. Methods Clin. Dev. 2, 15040 (2015). 26605372 10.1038/mtm.2015.40 PMC4632836

[60] C. Aurnhammer, M. Haase, N. Muether, M. Hausl, C. Rauschhuber, I. Huber, H. Nitschko, U. Busch, A. Sing, A. Ehrhardt, A. Baiker, Universal real-time PCR for the detection and quantification of adeno-associated virus serotype 2-derived inverted terminal repeat sequences. Hum. Gene Ther. Methods 23, 18–28 (2012). 22428977 10.1089/hgtb.2011.034

