# Supplementary material for: An AAV-CRISPR/Cas9 strategy for gene editing across divergent rodent species: Targeting neural oxytocin receptors as a proof of concept
Source: Sci Adv. 2023 May 31;9(22):eadf4950. doi: 10.1126/sciadv.adf4950 (PMC10413677; doi:10.1126/sciadv.adf4950)
Supplement: Supplementary file 1 — Fig. S1 [file sciadv.adf4950_sm.pdf]

Science Advances
AAAS

Supplementary Materials for

An AAV-CRISPR/Cas9 strategy for gene editing across divergent rodent species: Targeting neural oxytocin receptors as a proof of concept

Arjen J. Boender *et al.*

Corresponding author: Larry J. Young, lyoun03@emory.edu

Sci. Adv. **9**, eadf4950 (2023)
DOI: 10.1126/sciadv.adf4950

This PDF file includes:

Fig. S1

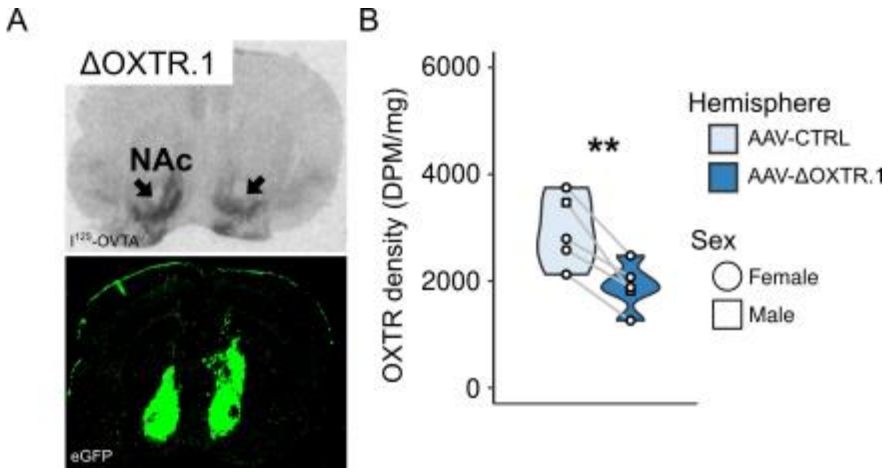

Figure S1. AAV-ΔOXTR.1 only partly reduces OXTR binding in spiny mice. (A) Upper image is a representative I^{125}-OVTA autoradiograms of a brain section of an AAV-CRISPR/Cas9-injected spiny mouse in the nucleus accumbens, and the lower image is an adjacent brain section that shows native eGFP-fluorescence. Black arrows indicate the target area (NAc = nucleus accumbens) **(B)** Quantification of OXTR levels in AAV-ΔOXTR-injected hemispheres and AAV-CTRL-injected hemispheres. **Paired t-test: N=5, P=0.005
